# Ultrasonic Solvent Extraction Followed by Dispersive Solid Phase Extraction (d-SPE) Cleanup for the Simultaneous Determination of Five Anthraquinones in *Polygonum multiflorum* by UHPLC-PDA

**DOI:** 10.3390/foods11030386

**Published:** 2022-01-28

**Authors:** Ying Xu, Xuan Yu, Jiaqi Gui, Yiqun Wan, Jinping Chen, Ting Tan, Fan Liu, Lan Guo

**Affiliations:** 1 College of Chemistry, Nanchang University, Nanchang 330047, China; 13517986724@163.com (Y.X.); whalien1214@163.com (X.Y.); jiaqigui@163.com (J.G.); wanyiqun@ncu.edu.cn (Y.W.); chenjinping2023@163.com (J.C.); 2 Center of Analysis and Testing, Nanchang University, Nanchang 330047, China; tanting@ncu.edu.cn (T.T.); fan_liu@ncu.edu.cn (F.L.); 3 Jiangxi Province Key Laboratory of Modern Analytical Science, Nanchang University, Nanchang 330047, China

**Keywords:** anthraquinones, *Polygonum multiflorum*, d-SPE, UHPLC

## Abstract

A rapid and effective ultra-high performance liquid chromatography (UHPLC) method was developed for the determination of five anthraquinones (emodin, physcion, aloe-emodin, rhein, and chrysophanol) in *Polygonum multiflorum*. The target compounds were ultrasonically extracted with 70% methanol, followed by dispersive solid-phase extraction (d-SPE) with HC-C18 and desorption with acetonitrile. The five anthraquinones were separated on an ACQUITY UPLC^®^ HSS T3 column (2.1 × 100 mm, 1.8 μm) and detected by a photodiode array detector (PDA) at 254 nm. Under the optimized conditions, linear relationships were achieved in the range of 0.3~100 mg/L for emodin, 0.3~40 mg/L for physcion, 0.1~20 mg/L for aloe-emodin, and 0.05~20 mg/L for rhein and chrysophanol. The limits of detection of the five analytes ranged from 0.01 to 0.08 mg/L, and the recoveries were within the range of 82.8~118.4% with an RSD (*n* = 6) of 1.0~10.3%. The intra-day and inter-day precision (*n* = 5) of the five targets were in the range of 1.0~1.8% and 3.0~3.1%, respectively. Furthermore, this method was applied to analyses of *Polygonum multiflorum* samples collected from different regions in China with satisfactory results. All the results indicated that this method is suitable for the detection of five anthraquinones in *Polygonum multiflorum*.

## 1. Introduction

*Polygonum multiflorum*, usually known as “Heshouwu” in China and “Foti” in North America and East Asia, is a perennial vine-like herb of the family Polygonaceae, and it was officially listed in Chinese Pharmacopoeia and noted 1200 years ago, in the Tang dynasty, for its medicinal values [1]. To date, numerous pharmacological studies and clinical trials have demonstrated the biological activities of *Polygonum multiflorum*, including hair blackening activities, memory improvement, mitigation against other dysfunctions, anti-tumor activities, anti-inflammatory activities, anti-oxidation activities, anti-aging activities, lipid-lowering activities, immune regulation, and so on [2,3,4,5,6,7]. Therefore, it has been widely used not only as a medicine, but also as a popular tonic food and beverage in Asia and many other countries as a result of the general population’s growing interests in phytonutrients and alternative medicines in recent decades [8]. Among the various constitutes of *Polygonum multiflorum*, stilbene glycosides and anthraquinones are considered to be the important indicators for quality control and content determination. Studies have shown that the antioxidant activity of *Polygonum multiflorum* was mainly attributed to stilbene glycosides [9], while anthraquinones proved to exhibit many biological activities, such as effects against cancer, developmental anomalies, and tonic tension [10].

However, some recent studies referring to the toxicity of *Polygonum multiflorum*, especially in adverse liver effects, have been reported [11,12,13]. Anthraquinones and their derivatives have been considered as the leading cause for the toxicity. It was found that anthraquinones exhibited severe toxicity to zebrafish embryos [12] and photoinduced acute toxicity in Daphnia magna [14]. Besides, the high cytotoxicity of anthraquinone derivatives such as rhein, emodin, and aloe-emodin has been found to be due to their potent binding affinity to DNA and glutathione [15]. Therefore, it is of great significance to establish an accurate, rapid, and reliable method to determine the anthraquinone components during the quality control and safety assessments of *Polygonum multiflorum*.

Common anthraquinones include emodin, physcion, aloe-emodin, rhein, chrysophanol, etc. Various analytical methods, such as high-performance liquid chromatography (HPLC) [16,17,18,19,20,21], capillary electrophoresis (CE) [22,23], gas chromatography (GC) [24], and high-performance thin-layer chromatography (HPTLC) [25] have been studied for the determination of anthraquinones in *Polygonum multiflorum* and other plant materials. Compared with other techniques, HPLC has played a major role in the determination of anthraquinones and other polar organic components in plants because it is not limited by the volatility or thermal stability of the analytes and demonstrates satisfactory sensitivity and reproducibility. However, the literature on the determination of anthraquinones in *Polygonum multiflorum* by liquid chromatography shows that the samples are usually treated by solvent reflux or ultrasonic extraction, and then the extractive is directly injected into the liquid chromatography system, without any cleanup procedure [16,17,18]. Although the sample pretreatment process is simple and fast, due to the absence of a purification process, the interference to the determination of target analytes and the damage to the chromatographic column brought by the coextracts of complex sample matrices cannot be ignored. 

The dispersive solid phase extraction technique (d-SPE), is a simple, quick, green, user-friendly, and cheap purification approach and has been widely used in the analysis of traditional Chinese medicines (TCMs) [26,27,28]. In this work, an environmentally friendly sample preparation method based on ultrasonic solvent extraction followed by d-SPE was developed and coupled with UHPLC-PDA for the determination of five anthraquinones in *Polygonum multiflorum*. The sample pretreatment protocol was discussed in detail and optimized for UHPLC-PDA analysis. Finally, the developed method was applied to the determination of anthraquinones in real *Polygonum multiflorum* samples successfully.

## 2. Materials and Methods

### 2.1. Reagents and Materials

The standards of emodin, chrysophanol, physcion, rhein, aloe-emodin (purity, HPLC ≥ 98%), silica gel, and silicon dioxide were purchased from Aladdin Biochemical Technology Co., Ltd. (Shanghai, China). Methanol and acetonitrile (HPLC grade) were purchased from TEDIA (Anhui, China). Formic acid (HPLC grade) was purchased from Anaqua Chemical Supply Inc (Wilmington, NC, USA). Primary secondary amine (PSA) was purchased from Bangkai High-tech Materials Company (Qingdao, China). HC-C18 was purchased from ANPLE Company (Shanghai, China). Zeolite was purchased from Energy Chemical Company (Shanghai, China). Ultra-pure water was prepared by the PULSE-Analytic ultra-pure water system (ELGA Company, High Wycombe, UK).

Stock standard solutions of emodin, physcion, rhein, chrysophanol, and aloe-emodin were prepared at 1000 mg/L with methanol separately. The standards were kept at 4 °C. Mixed, working-standard solutions were prepared by dilution with methanol to get the required concentration.

### 2.2. Chromatographic Conditions

UHPLC analysis was performed on a Waters Acquity UPLC H-Class Plus system (Waters Company, Milford, MA, USA) equipped with a photodiode array detector (PDA) and an ACQUITY UPLC^®^ HSS T3 column (2.1 × 100 mm, 1.8 μm). Chromatographic separation was carried out at 35 °C. The mobile phase comprised solvent A (0.1% (*v*/*v*) formic acid aqueous solution) and solvent B (0.1% (*v*/*v*) formic acid in acetonitrile). The gradient elution program was shown on Table 1. The flow rate was 0.4 mL/min and the injection volume was 5 μL. The monitoring wavelength was set at 254 nm for five anthraquinones. 

### 2.3. Water Content Determination

The *Polygonum multiflorum* samples were meshed and sieved through 80 mesh. Between 2~5 g (accurately weighed to 0.0001 g) of ground sample powder was weighed and placed in a flat weighing bottle which had been dried to constant weight; the powder thickness was no more than 5 mm. Then, it was dried in the weighing bottle at 100–105 °C for 5 h with the cap opened. After drying, the cap was closed, the bottle was transferred to a dryer, it was cooled for 30 min, and then weighed. Afterward, it was dried again at 100–105 °C for 1 h, cooled for 30 min, and weighed until the difference in mass between two consecutive measurements was no more than 5 mg. According to the weight loss, the water content (%) of *Polygonum multiflorum* sample was calculated.

### 2.4. Sample Preparation

A total of 0.5 g of ground sample powder was weighed in a 50 mL polypropylene centrifuge tube using an Adventurer electronic balance (Ohaus Company, Shanghai, China) and then 30.0 mL of 70% methanol was added to the tube. After ultrasonic extraction at 100 W power for 30 min with a KQ3200DE CNC ultrasonic cleaning machine (Kunshan Ultrasonic Instrument Co., Ltd., Kunshan, China), the mixture was centrifuged at 4555× *g* for 5 min with a TG20.5 high speed centrifuge (Shanghai Lu Xiangyi Centrifuge, Shanghai, China). Then, 10 mL of supernatant was taken and diluted with 4 mL of water to make the concentration of methanol in the supernatant 50%. 

Then, 0.6 g of HC-C18 was added to 5 mL of the above extraction solution, and the mixture was vortexed vigorously for 10 min at 3000 rpm with a MS3 mini shaker (Guangzhou Yike Lab Technology LTM Co., Guangzhou, China) to adsorb the analytes. The adsorbent was separated and collected by centrifuging at 10,275× *g* for 10 min. The above adsorption steps were repeated twice and all HC-C18 adsorbent was collected. 

Finally, the analytes adsorbed on the HC-C18 were eluted with 20.0 mL of acetonitrile by ultrasonic for 30 min. A total of 6.0 mL of the acetonitrile was taken and dried with nitrogen. The solid residue was dissolved with 150 μL of methanol and filtered through a 0.22 μm organic filter membrane for UHPLC analysis.

## 3. Results and Discussion

### 3.1. Optimization of Chromatographic Conditions

To obtain satisfactory separation of the five anthraquinones, three mobile phase systems, including water-acetonitrile, 0.1% (*v*/*v*) formic acid aqueous solution-acetonitrile, and 0.1% (*v*/*v*) formic acid aqueous solution-0.1% (*v*/*v*) formic acid in acetonitrile were investigated. The experimental results showed that the peak shape could be obviously improved when formic acid was used as a mobile phase modifier. The best separation efficiency and peak shapes were obtained when 0.1% (*v*/*v*) formic acid aqueous solution-0.1% (*v*/*v*) formic acid in acetonitrile was applied as the mobile phase. Afterward, the gradient elution procedures were optimized. The chromatographic separation was conducted on an ACQUITY UPLC^®^ HSS T3 column with a gradient elution program (as shown on Table 1) at a flow rate of 0.4 mL/min, and the column temperature was set at 35 °C. Considering the UV absorption spectrum characteristics of the five targets, the determination wavelength was set at 254 nm, which could obtain high UV absorption signals to ensure the sensitivity of detection.

**Figure 1:**
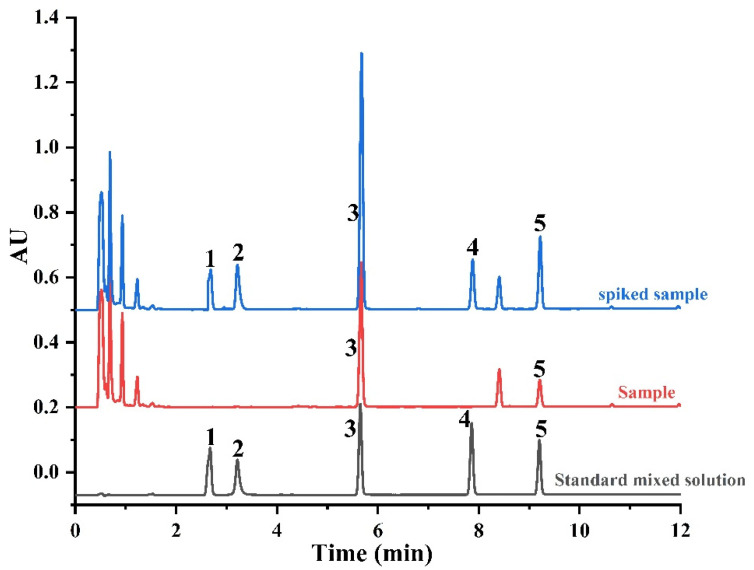
A chromatogram of the standard mixed solution, *Polygonum multiflorum* sample, and spiked *Polygonum multiflorum*. The identified peaks are: (1) aloe-emodin; (2) rhein; (3) emodin; (4) chrysophanol; and (5) physcion.

Under optimized chromatographic conditions, the chromatogram of the five anthraquinones in standard solution is shown in Figure 1. This demonstrates the good chromatographic separation possible within 10 minutes. The mixed standard solution was analyzed six times: the retention times of aloe-emodin, rhein, emodin, chrysophanol, and physcion were 2.68 ± 0.03, 3.27 ± 0.05, 5.69 ± 0.08, 7.89 ± 0.08, and 9.24 ± 0.10 min, respectively.

The target compounds in the sample were identified by comparing the retention time and the UV spectrum with that in the standard solution. The chromatograms of a sample and a spiked sample were also shown in Figure 1. It can be seen that the five anthraquinones all have the same retention times in the standard mixed solution, sample, and spiked sample, and the compounds introduced by the sample matrix obtained baseline separation from the five anthraquinones, meaning that under these conditions, the matrix had no effect on the separation and determination of the target compounds.

### 3.2. Optimization of the Ultrasonic Extraction Conditions

Among the extraction methods of Chinese herbal medicine, ultrasonic extraction is a commonly used method. The ultrasonic cavitation formed during the ultrasonic-assisted extraction process can instantly complete the rupture of plant cell wall. At the same time, the frequency and speed of molecular movement were accelerated significantly, which greatly accelerated the transfer of the target analyte from the cell to the solvent, so the extraction efficiency was also significantly improved. Therefore, in our work, the ultrasonic extraction method was used to extract the five anthraquinones from *Polygonum multiflorum*.

To optimize the ultrasonic extraction conditions, the effects of the type of extraction solvent, the amount of extraction solvent, and the extraction time, which affect the extraction efficiency, were investigated, using *Polygonum multiflorum* as sample. Since large amounts of emodin and physcion were contained in the *Polygonum multiflorum* samples, the extracted amounts of emodin and physcion were used to compare the real extraction efficiency of the analytes under different conditions.

**Figure 2:**
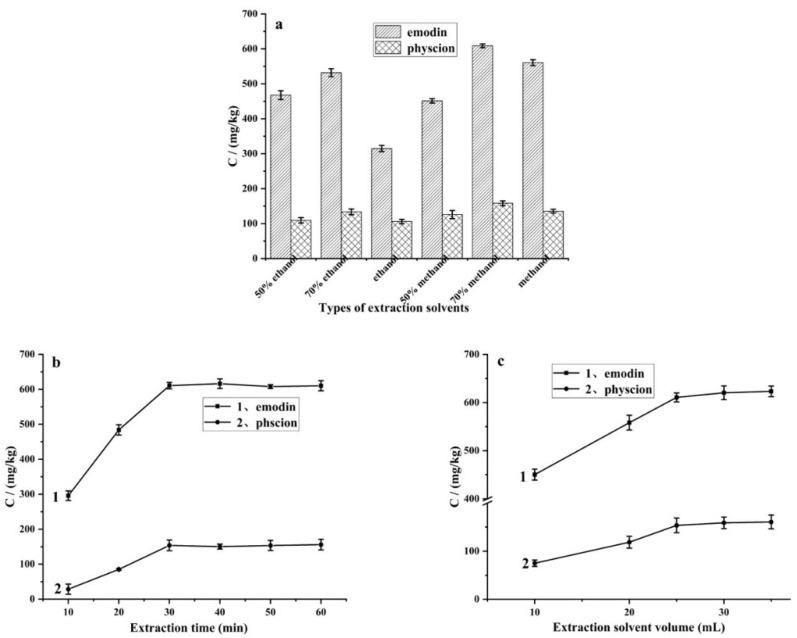
The effect of the type of extraction solvents (**a**), extraction time (**b**), and volume of extraction (**c**) on the extraction efficiency. (*n* = 3) Conditions: (**a**) Volume of extraction solvent, 25 mL; extraction time, 60 min. (**b**) Extraction solvent, 70% methanol; volume of extraction solvent, 25 mL. (**c**) Extraction solvent, 70% methanol; extraction time, 30 min.

First, six solvents (50% ethanol, 70% ethanol, ethanol, 50% methanol, 70% methanol and methanol) were used separately as the extraction solvent. As shown in Figure 2a, methanol displayed a higher extraction efficiency than ethanol, and the best extraction efficiencies of emodin and physcion were achieved by using 70% methanol. So, 70% methanol was selected as the extraction solvent in the following experiments. 

Then, in order to further increase the extraction efficiency, the extraction time in the range of 10–60 min was optimized. As shown in Figure 2b, the amount of emodin and physcion extracted from the sample increased as the extraction time increased, and reached equilibrium after 30 min. 

The amount of extraction solvent also demonstrates an important influence on the extraction efficiency. In this study, when the mass of the sample is 0.5 g, the effects of the solvent amount in the range of 10–35 mL on the extraction efficiency were investigated. The results (Figure 2c) showed that the amount of emodin and physcion increased when the solvent volume increased from 10 mL to 25 mL, and then balanced when further increasing the solvent volume. Therefore, the samples were ultrasonically extracted by 30 mL of 70% methanol aqueous solution for 30 min in this study.

In order to further validate the ultrasonic extraction method, a comparison was conducted between ultrasonic extraction and Soxhlet extraction. The procedure for Soxhlet extraction was as follows: 0.5 g of sample powder was weighed in the Soxhlet extraction tube, then extracted with 50.0 mL of methanol for 8 h. The results are listed in Table 2. Table 2 shows that the contents of emodin and physcion of the two extraction methods were equivalent, indicating that the extraction efficiency of ultrasonic extraction was acceptable. Furthermore, ultrasonic extraction can greatly shorten the extraction time and reduce the amount of extraction solvent.

### 3.3. Optimization of the Adsorption Conditions

In order to reduce the interference to the determination and the damage to the chromatographic column caused by the coextracts of the complex sample matrix, the d-SPE technique was applied as a cleanup procedure. Some materials can be used as d-SPE adsorbents to purify the extraction solution prior to UHPLC analysis. To optimize the adsorption conditions, the effects of the type of adsorption material, the methanol content in the extraction solution, the adsorption time, and the amount of adsorbent, all of which affect the adsorption efficiency, were investigated in this study, using the standard solution as a sample. The adsorption experiment process was as follows: 5 mL of 10 mg/L mixed standard solution was taken as the initial solution, the original standard mass m_0_ was calculated, an adsorbent was used to adsorb the standards, and the remained standards (m_1_) were centrifugated and quantified in the supernatant. The calculation formula of adsorption efficiency was as follows:

$$[(m_{0}\;-\;m_{1})/m_{0}]\;\times \;100\%$$

**Figure 3:**
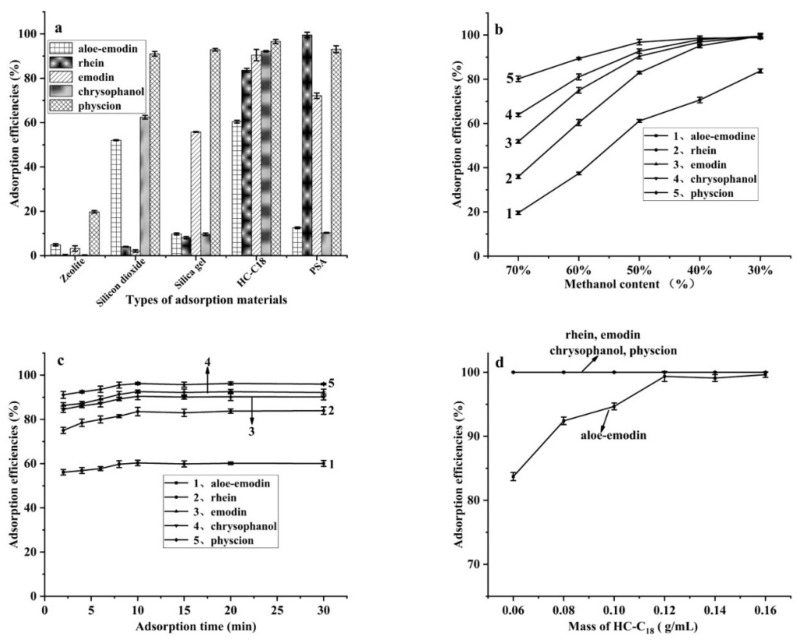
The effects of the type of adsorption materials (**a**), methanol content (**b**), adsorption time (**c**), and the mass of the adsorbents (**d**) on five anthraquinones in mixed standard solution. (*n* = 3) Conditions: (**a**) Initial standard solution concentration, 10 mg/L each; methanol content, 50%; adsorption time, 60 min; volume of standard solution, 5 mL; HC-C18 (0.10 g/mL). (**b**) Initial standard solution concentration, 10 mg/L each; adsorption material, HC-C18 (0.10 g/mL); adsorption time, 60 min; volume of standard solution, 5 mL. (**c**) Initial standard solution concentration, 10 mg/L each; methanol content, 50%; HC-C18 (0.10 g/mL); volume of standard solution, 5 mL. (**d**) Initial standard solution concentration, 10 mg/L each; methanol content, 50%; volume of standard solution, 5 mL; adsorption time, 10 min. Each content was adsorbed three times.

The adsorption efficiency of the five commercial adsorbents (zeolite, silica gel, silicon dioxide, primary secondary amine (PSA), and HC-C18) on the anthraquinones was investigated. As shown in Figure 3a, the yields of the five anthraquinones with HC-C18 are obviously higher than those with other materials. This result may be contributed to the high carbon content and high hydrophobicity of HC-C18. Therefore, HC-C18 was selected as the adsorbent in the following experiments.

The methanol content in the solution changes the polarity of the solution, and thus may affect the interaction of the targets with the HC-C18 adsorbent. Therefore, the influence of methanol content on the adsorption efficiency was investigated by varying the methanol content in the range of 30–70%. As shown by the results in Figure 3b, the adsorption efficiency increased as the methanol content decreased from 70% to 30%. However, when the methanol content was lower than 50%, the anthraquinones were partly precipitated from the methanol solution after being placed at room temperature for 1 h, so the methanol content was finally selected as 50%. 

In order to obtain favorable adsorption efficiency and shorten analysis time, the adsorption time was optimized in the range of 2–30 min. As shown by the results in Figure 3c, the adsorption efficiency increases as the adsorption time increases and reached equilibrium after 10 min. Therefore, in the subsequent experiments, the adsorption time was set as 10 min.

The effects of the amount of HC-C18 on the adsorption efficiencies of the five analytes were investigated in the range of 0.06–0.16 g/mL. As shown in Figure 3d, after the third adsorption, aloe-emodin can be completely adsorbed when more than 0.12 g/mL of HC-C18 is used, and the other four analytes can be completely adsorbed when 0.06 g/mL of HC-C18 is used. In the subsequent experiments, 0.12 g/mL of HC-C18 was used and the above adsorption steps were repeated three times.

### 3.4. Optimization of the Desorption Conditions

To optimize the desorption conditions, the effects of desorption solvent (type and volume) and the desorption time were investigated. The desorption experiment process was as follows: desorption solvent was used to elute the standards from adsorbent HC-C18 and the standard (m_2_) was centrifugated and quantified in the elution. The calculation formula of desorption efficiency was as follows:

$$[(m_{2})/(m_{0}\;-\;m_{1})]\;\times \;100\%$$

**Figure 4:**
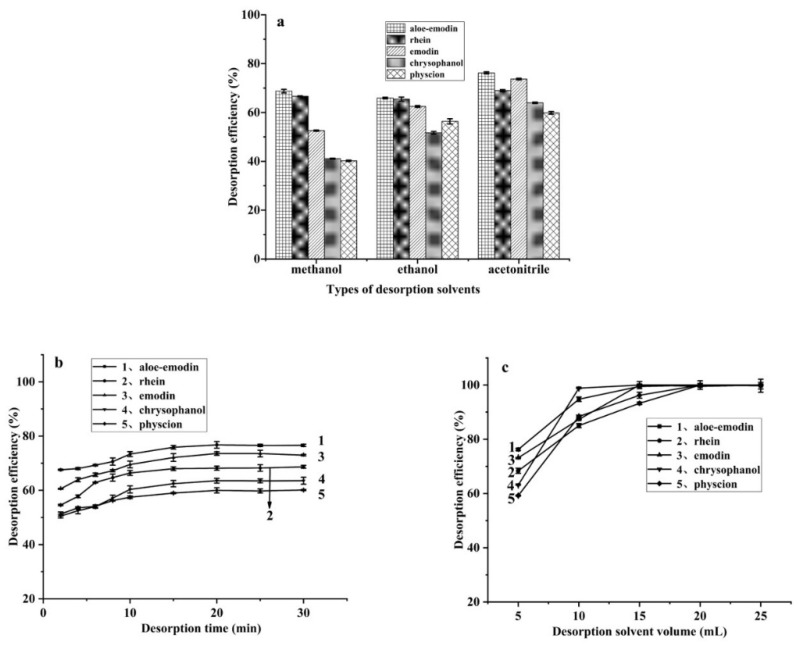
The effect of the type of desorption solvents (**a**), desorption time (**b**), and the volume of desorption (**c**). (*n* = 3) Conditions: (**a**) Initial standard solution concentration, 10 mg/L each; methanol content, 50%; volume of standard solution, 5 mL; adsorption time, 10 min; HC-C18 (0.12 g/mL) (adsorption 3 times); volume of desorption solvent, 5 mL; desorption time, 30 min. (**b**) Initial standard solution concentration, 10 mg/L each; methanol content, 50%; volume of standard solution, 5 mL; adsorption time, 10 min; HC-C18 (0.12 g/mL) (adsorption 3 times); desorption solvent, acetonitrile; volume of desorption solvent, 5 mL. (**c**) Initial standard solution concentration, 10 mg/L; methanol content, 50%; volume of standard solution, 5 mL; adsorption time, 10 min; HC-C18 (0.12 g/mL) (adsorption 3 times); desorption solvent, acetonitrile; desorption time, 30 min.

A suitable desorption solvent plays a vital role in the desorption experiment, and it is a pivotal factor affecting the elution effect. The influence of different desorption solvents on the five analytes was investigated with methanol, ethanol, and acetonitrile. As shown in Figure 4a, acetonitrile displayed higher desorption efficiencies for the five anthraquinones than methanol and ethanol. Therefore, acetonitrile was used as the desorption solvent in the following studies.

To obtain the best desorption efficiencies, the desorption time was optimized in the range of 2–40 min. As shown in Figure 4b, the desorption efficiency of the five anthraquinones increased as the desorption time increased from 2–20 min, and then kept balance when the time was increased further. Therefore, in the subsequent experiments, the desorption time was set as 25 min. 

The volume of desorption solvent also has important effects on desorption efficiency. The volume of desorption solvent was optimized in the range of 5–25 mL. The results (Figure 4c) showed that the desorption efficiencies of the five analytes increased when the solvent volume increased from 5 mL to 20 mL. When more than 20 mL of acetonitrile was used, the desorption efficiencies changed slightly. Therefore, 20 mL of acetonitrile was used to elute the analytes in this study.

### 3.5. Method Validation

In order to determine the linearity of the five anthraquinones, a series of mixed standard solutions were prepared and measured under the optimized conditions. The linear regression equations were obtained by performing a linear regression on the analyte concentrations (x) with peak area (y). The mixed standard solution with 0.2 mg/L of emodin and physcion and 0.05 mg/L of aloe-emodin, rhein, and chrysophanol were continuously injected 11 times, and the standard deviation (SD) of the five analytes were calculated. The limit of detection (LOD) and limit of quantification (LOQ) were calculated by 3 SD/*m* and 10 SD/*m* (where *m* is the slope of the calibration line), respectively. The results in Table 3 showed that linear relationships (r ≥ 0.9982) were achieved in the range of 0.3~100 mg/L for emodin, 0.3~40 mg/L for physcion, 0.1~20 mg/L for aloe-emodin, and 0.05~20 mg/L for rhein and chrysophanol. The LODs and LOQs of the five targets were in the range of 0.01~0.08 mg/L and 0.04~0.28 mg/L, respectively. The intra-day and inter-day precision tests of the analytical platform were performed with a mixed standard solution of 10 mg/L. Each experiment was repeated five times. The intra-day and inter-day precision of the five targets were in the range of 1.0~1.8% and 3.0~3.1%, respectively.

In order to further evaluate the accuracy and precision of the method, recovery experiments with three spiked concentration levels were carried out under the optimized conditions. An appropriate amount of standard mixed solution was added to the *Polygonum multiflorum* sample powder and the sample was allowed to stand for 2 h to allow the standard to fully react with the matrix. Then, the contents of the five targets in the sample and the spiked sample were determined and the recoveries were calculated. The average recoveries and relative standard deviation (RSD) (*n* = 6) of each compound are presented in Table 4. The average recoveries of the five anthraquinones were in the range of 82.8–118.4% with RSDs (*n* = 6) ranging from 1.0% to 10.3%. These results indicate that this method is accurate and reliable and can be used to determine the five anthraquinones in *Polygonum multiflorum*.

### 3.6. Sample Determination

**Figure 5:**
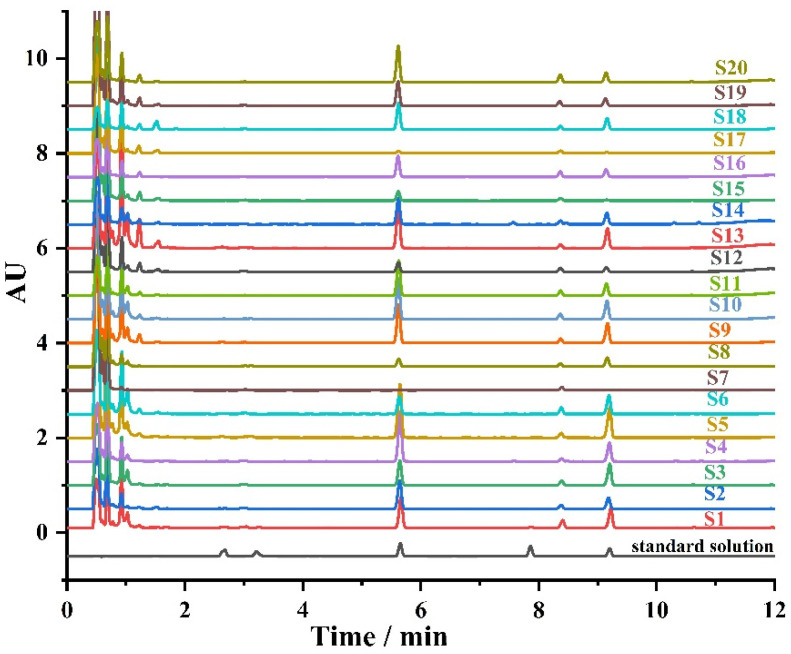
The chromatograms of the 20 *Polygonum multiflorum* samples.

The 20 Polygonum multiflorum samples collected from different regions in China were analyzed by the method developed above. The chromatograms of the 20 samples are shown in Figure 5, and the determined results are shown in Table 5. The water content was in the range of 3.8~9.5 %, which is in line with the requirements for water content should not exceed 10% in Chinese Pharmacopoeia. (≤10.0%). The content of five anthraquinones in Polygonum multiflorum samples were calculated according to the dry product. The determined results of emodin, physcion, aloe-emodin, rhein, and chrysophanol were in the range of 55.5~1100.4, 42.4~348.3, 0~18.3, 0~22.6, and 0~85.4 mg/kg, respectively. 

The results show that the contents of emodin and physcion, which are often used as quality control indexes of Polygonum multiflorum, were significantly higher than that of the other three anthraquinones in the same sample. In addition, there were also large differences in the content of the same component in Polygonum multiflorum samples from different regions. This may be due to the differences of soil, climate, and photoperiod in different regions.

### 3.7. Comparison with Other Methods

The analysis performance of our method was compared with that of some reported methods for the determination of anthraquinones in *Polygonum multiflorum* samples. The results are listed in Table 6. Our method could determine five anthraquinones while the literature could only measure two anthraquinones, emodin and physcion. The determination results of 20 *Polygonum multiflorum* samples collected from different regions of China, as listed on Table 4, show that emodin and physcion are indeed the main components of anthraquinones in *Polygonum multiflorum*, but three other anthraquinones, aloe-emodin, rhein, and chrysophanol, also exist in *Polygonum multiflorum*, the content of which, in some samples, was not low, even reaching 85 mg/kg. Therefore, it is necessary to establish a method for the simultaneous determination of the five anthraquinones in *Polygonum multiflorum*. Moreover, compared with other methods, our work added a d-SPE purification step after the extraction procedure, which could effectively reduce the interference in determination and protect the chromatographic column. In terms of the common target compounds emodin and physcion, the detection limits of our method were not as good as that of HPLC-MS but were equivalent to that of HPLC-UV. Therefore, this work is suitable for the determination of the five anthraquinones in *Polygonum multiflorum*.

## 4. Conclusions

A simple and effective UHPLC method was developed and validated for the simultaneous determination of five anthraquinones (emodin, physcion, chrysophanol, rhein and aloe-emodin) in *Polygonum multiflorum*. The procedure began with sample ultrasonic extraction using 70% methanol, followed by d-SPE purification using an HC-C18 adsorbent prior to analysis by UHPLC. The average recoveries were in the range of 82.8–118.4% with RSDs (*n* = 6) less than 10.3%. The correlation coefficients of the calibration curves exceeded 0.9982 in the linear range. The anthraquinone species detected by this method were more than that of the previous HPLC studies of *Polygonum multiflorum* [16,17,18], moreover, the d-SPE cleanup procedure could reduce the interference to the analysis and the damage to the chromatographic column. Further studies demonstrated that this method could be effectively applied to the analysis of five anthraquinones in *Polygonum multiflorum*. The determination results of 20 *Polygonum multiflorum* samples collected from different regions of China showed that emodin and physcion were the main components of anthraquinones in *Polygonum multiflorum*, but three other anthraquinones, aloe-emodin, rhein, and chrysophanol, also existed in *Polygonum multiflorum*. In addition, there were large differences in the content of the same component in *Polygonum multiflorum* samples from different regions. 

## Figures and Tables

**Table 1:** The gradient elution conditions.

Time (min)	A (%)	B (%)
0	55	45
1	55	45
10	30	70
13	10	90
13.1	55	45
15	55	45

**Table 2:** A comparison of ultrasonic extraction and Soxhlet extraction (*n* = 3).

Extration Method	Emodin (mg/kg)	Physcion (mg/kg)
Soxhlet extraction	625.30 ± 2.36	160.09 ± 3.71
Ultrasonic extraction	616.35 ± 3.24	158.96 ± 1.58

**Table 3:** The linear equations, correlation coefficients, precision, limits of detection (LODs, 3SD/*m*), and limits of quantification (LOQs, 10 SD/*m*) for emodin, physcion, aloe-emodin, rhein, and chrysophanol.

Compounds	Linear Range (mg/L)	Linear Equations	Correlation Coefficients (r)	LODs (mg/L)	LOQs (mg/L)	Intra-Day Precision (RSD, %, *n* = 5)	Inter-Day Precision (RSD, %, *n* = 5)
Emodin	0.3–100	y = 63161.74x − 10223.95	0.9982	0.07	0.23	1.5	3.0
Physcion	0.3–40	y = 40140.68x + 703.33	0.9992	0.08	0.28	1.0	3.0
Aloe-emodin	0.1–20	y = 74506.30x − 1424.71	0.9999	0.02	0.07	1.6	3.0
Rhein	0.05–20	y = 52340.46x + 601.59	0.9998	0.01	0.04	1.8	3.1
Chrysophanol	0.05–20	y = 82185.20x − 3521.00	0.9995	0.02	0.05	1.5	3.0

**Table 4:** The recovery and precision for the determination of the five anthraquinones (*n* = 6).

Sample	Compounds	Original (mg/kg)	Added (mg/kg)	Measured (mg/L)	Found (mg/kg)	Average Recovery (%)	RSD (%)
*Polygonum multiflorum*	Emodin	454.22	170 250 340	76.68 84.66 93.98	644.11 711.14 789.43	111.7 102.8 98.6	4.6 1.8 6.7
	Physcion	113.41	40 85 125	19.14 25.12 28.07	160.78 211.01 235.79	118.4 114.9 97.9	6.2 1.1 1.0
	Aloe-emodin	ND	1.70 8.50 85.0	0.22 1.02 10.49	1.86 8.53 88.16	109.5 100.4 103.7	10.3 2.3 5.1
	Rhein	ND	1.70 8.50 85.0	0.21 1.03 9.71	1.80 8.68 81.56	105.8 102.1 96.0	2.2 1.9 4.4
	Chrysophanol	ND	1.70 8.50 85.0	0.23 0.84 9.44	1.94 7.04 79.30	114.1 82.8 93.3	9.3 1.8 5.0

ND: not detected or lower than LOD.

**Table 5:** The results of UHPLC-PDA analysis of 20 *Polygonum multiflorum* samples collected from different regions in China.

Sample	Origin	Water Content (%)	Content mg/kg (*n* = 3)				
			Emodin	Physcion	Aloe-Emodin	Rhein	Chrysophanol
1	Sichuan	9.3	605.8 ± 1.5	312.1 ± 2.3	ND	ND	ND
2	Sichuan	7.8	641.9 ± 2.5	248.7 ± 0.3	12.9 ± 0.1	ND	ND
3	Sichuan	4.8	436.4 ± 1.2	238.5 ± 0.8	ND	ND	ND
4	Yunnan	8.4	867.4 ± 3.6	315.9 ± 0.8	6.0 ± 0.1	ND	ND
5	Yunnan	6.5	1100.4 ± 11.9 *	256.6 ± 3.8	1.9 ± 0.4	22.6 ± 0.3	ND
6	Yunnan	3.8	349.8 ± 3.9	347.7 ± 0.5	18.3 ± 0.3	ND	ND
7	Guizhou	4.7	55.5 ± 0.2	42.4 ± 0.4	ND	ND	ND
8	Guizhou	4.6	170.0 ± 1.3	188.4 ± 1.5	ND	12.0 ± 0.2	ND
9	Hubei	8.3	591.5 ± 4.3	300.4 ± 1.6	ND	ND	ND
10	Hubei	8.6	685.8 ± 4.7	306.1 ± 1.0	ND	7.6 ± 0.1	ND
11	Anhui	8.6	564.7 ± 6.1	205.7 ± 1.7	1.4 ± 0.1	ND	ND
12	Shanxi	9.1	186.0 ± 1.4	103.9 ± 0.5	ND	ND	ND
13	Guangxi	9.5	835.7 ± 5.0	348.3 ± 3.0	ND	3.7 ± 0.2	ND
14	Guangxi	7.5	403.6 ± 3.0	202.3 ± 0.5	ND	ND	16.8 ± 0.1
15	Jiangsu	6.0	159.1 ± 0.2	55.4 ± 0.1	ND	1.3 ± 0.1	85.4 ± 0.5
16	Guangdong	5.2	337.1 ± 2.4	143.3 ± 0.9	ND	ND	ND
17	Jiangxi	7.9	85.3 ± 1.3	56.0 ± 0.4	ND	ND	ND
18	Shandong	7.7	448.8 ± 1.5	189.3 ± 2.2	ND	3.4 ± 0.2	ND
19	Chongqing	5.2	417.0 ± 4.3	143.2 ± 0.9	ND	ND	ND
20	Henan	4.9	556.0 ± 7.1	163.0 ± 2.7	ND	ND	ND

ND: not detected or lower than LOD. *: The value was obtained after dilution of the sample.

**Table 6:** A comparison of reported methods with our work.

Sample	Analytical Method	Extraction Technology	Purification Method	Species	LODs (mg/L)	Ref
*Polygonum multiflorum*	HPLC-MS	Ultrasonic extraction	/	Emodin, Physcion	0.00063~0.00082	[16]
*Polygonum multiflorum*	LC-MS/MS	Ultrasonic extraction	/	Emodin, Physcion	0.0007~0.008	[17]
*Polygoni Multiflori Caulis*	HPLC-UV	Ultrasonic extraction	/	Emodin, Physcion	0.04~0.06	[18]
*Polygonum multiflorum*	UHPLC-PDA	Ultrasonic extraction	Dispersive solid phase extraction	Aloe-emodin, Rhein, Emodin, Chrysophanol, Physcion	0.01~0.08	Our work

## Data Availability

Data is contained within the article.
